# *In Vivo* and *In Vitro* Matured Oocytes From Mice of Advanced Reproductive Age Exhibit Alternative Splicing Processes for Mitochondrial Oxidative Phosphorylation

**DOI:** 10.3389/fendo.2022.816606

**Published:** 2022-01-26

**Authors:** Hao Qin, Yi Qu, Rong Li, Jie Qiao

**Affiliations:** ^1^ Center for Reproductive Medicine, Department of Obstetrics and Gynecology, Peking University Third Hospital, Beijing, China; ^2^ National Clinical Research Center for Obstetrics and Gynecology (Peking University Third Hospital), Beijing, China; ^3^ Key Laboratory of Assisted Reproduction (Peking University), Ministry of Education, Beijing, China; ^4^ Beijing Key Laboratory of Reproductive Endocrinology and Assisted Reproductive Technology, Beijing, China; ^5^ Research Units of Comprehensive Diagnosis and Treatment of Oocyte Maturation Arrest, Chinese Academy of Medical Sciences, Beijing, China

**Keywords:** oocyte, *in vitro* maturation, oxidative phosphorylation, transcriptome, differentially expressed genes, alternative splicing

## Abstract

The mean age of women seeking infertility treatment has gradually increased over recent years. This has coincided with the emergence of *in vitro* maturation (IVM), a method used in assisted reproductive technology for patients with special requirements. However, when compared with conventional *in vitro* fertilization, IVM is associated with poor embryonic development potential and low live birth rates, thus limiting the widespread application of this technique. In this study, we performed RNA-sequencing transcriptomic assays and identified a total of 2,627 significant differentially expressed genes (DEGs) between IVM oocytes and *in vivo* matured oocytes from mice of advanced reproductive age. Next, Kyoto Encyclopedia of Genes and Genomes pathway analysis was used to identify the potential functions of the DEGs. The most significantly enriched pathway was oxidative phosphorylation (OXPHOS). In addition, we constructed a protein-protein interaction network to identify key genes and determined that most of the hub genes were mtDNA-encoded subunits of respiratory chain complex I. Antioxidant supplementation lead to an increase in ATP production and reduced the gene expression profile of the OXPHOS pathway in the IVM group. Moreover, alternative splicing (AS) events were identified during *in vivo* or *in vitro* oocyte maturation; data showed that skipped exons were the most frequent type of AS event. A number of genes associated with the OXPHOS pathway exhibited alterations in AS events, including *Ndufa7*, *Ndufs7*, *Cox6a2*, *Ndufs5*, *Ndufb1*, and *Uqcrh*. Furthermore, the process of IVO promoted the skipping of exon 2 in *Ndufa7* and exon 3 in *Ndufs7* compared with the IVM oocytes, as determined by semi−quantitative RT−PCR. Collectively, these findings provide potential new therapeutic targets for improving IVM of aged women who undergo infertility treatment.

## Introduction

Global fertility has declined significantly over recent years and this has led to infertility becoming the third most prevalent disease after tumors and cardiovascular disease (1). The decline in fertility caused by advanced maternal age (≥ 35 years) has led to a significant increase in the incidence of infertility. Assisted reproductive technology (ART) offers a significant hope of pregnancy to women who are suffering from infertility. Notably, the mean age of women undergoing ART treatment has gradually increased (2, 3). *In vitro* maturation (IVM) of oocytes has emerged as an important ART procedure involving the *in vitro* culture and offers an alternative option for women with polycystic ovary syndrome (4). IVM can also prevent ovarian hyperstimulation syndrome during ovarian stimulation (5), can be of benefit to poor responders (6), and can help preserve fertility in patients being treated for cancer (7). However, previous studies have reported that maternal aging is associated with reduced IVM success rates and adverse events (8, 9). IVM is an alternative option for ovarian tissue cryopreservation (OTC); however, the success rates for IVM can be very low when used to mature oocytes retrieved from females of advanced age during OTC (10). Therefore, there is an urgent need to identify the molecular mechanisms underlying IVM so that this technique can be become a driving force for the implementation of ART technology.

The process of mitochondrial oxidative phosphorylation (OXPHOS) is a major metabolic pathway associated with ATP generation and involves five major membrane complexes (11). The OXPHOS system is the major source of reactive oxygen species (ROS) in mitochondria (12). Previous studies have revealed that the production of ROS *via* mitochondrial metabolism was higher in IVM oocytes than *in vivo* matured (IVO) oocytes (13, 14). This difference may be attributed to a number of environmental factors, including light exposure and the presence of antioxidants in the follicular fluid (15–17). In a previous study, Sanfins et al. reported that the fertilization rate was lower, and embryo development was delayed, in IVM oocytes than in IVO oocytes; these authors also reported a difference between these two types of oocytes with regards to cytoskeleton organization and nuclear lamina integrity (18, 19). Previous research identified several differentially expressed genes (DEGs) between IVM and IVO oocytes and demonstrated that the GATA-1/CREB1/WNT pathway is associated with the quality of human oocytes (20) and detected the cellular metabolism and genetic alterations in mouse oocytes (21). Furthermore, in our previous study, we investigated dynamic changes of gene expression between human IVM and IVO oocytes using single-cell transcriptome sequencing analysis. We found, for the first time, that metabolic function was impaired in human oocytes maintained in the *in vitro* environment (22). Our present study demonstrates, for the first time, the existence of altered alternative splicing (AS) events in the mitochondrial OXPHOS pathway between IVM and IVO oocytes from mice of advanced reproductive age.

Precursor mRNA refers to the process in which exons and introns are either excluded or included in gene expression, thus generating multiple different mature mRNAs and resulting in increased protein diversity (23, 24). A previous study revealed that approximately 95% of multi-exonic genes undergo AS regulation in humans, as determined by high-throughput sequencing technology (25). This regulatory system can be commonly categorized into five canonical patterns including skipped exon (SE), mutually exclusive exon (MXE), retained intron (RI), alternative 3´ and 5´ splice site (A3SS and A5SS) that are associated with the regulation of cell lineage differentiation, subcellular localization, tumor progression, and germ cell development (26–29). Furthermore, numerous reports have indicated that the aberrant pre-mRNA splicing of functional genes is involved in abnormal spermatogenesis and male infertility (30, 31). In mammalian oocytes, conserved stage-specific transcript variants indicate that alternative pre-mRNA splicing plays a crucial role in the accurate control of the maternal transcriptome (32, 33). For example, ESRP1 is involved in the AS programming of oocyte mRNA processing and the ESRP1 deletion-induced pre-mRNA splicing changes in spindle organization-related genes that lead to female infertility (34). Depletion of the nuclear m6A reader YTHDC1 causes extensive alternative polyadenylation and the 3′-UTR length is altered in oocytes, thus resulting in female sterility (35). Previous work in our group demonstrated that AS events are important regulatory mechanisms associated with the failure of maturation in human oocytes (36). However, our understanding of the differential expression of pre-mRNA splicing during the maturation of mammalian oocytes remains largely obscure.

In the present study, we used mice of advanced reproductive age as oocyte donors and identified differences in gene regulation during oocyte maturation when comparing IVO and IVM oocytes. Next, we investigated the molecular mechanisms underlying these changes at the level of the transcriptome. We performed KEGG pathway enrichment analysis and found that mitochondrial OXPHOS was significantly enriched. We also constructed an interaction network for the DEGs to investigate interacting partners and identified differential AS events among the DEGs that implied the potential physiological regulatory mechanisms may account for the inadequacies of oocytes derived by IVM. This research provides new insights into the outcomes of ART for women of advanced age with deficits in oocyte quality. Furthermore, we identified potential therapeutic biomarkers for clinical diagnosis that can be applied during the development of IVM oocytes.

## Materials and Methods

### Mice

All animal procedures were approved by the Institutional Animal Care and Use Committee of Peking University. Female Institute of Cancer Research (ICR) mice at the age of 42-45 weeks (reproductively old mice) were purchased from Charles River Laboratories (Beijing, China) and were maintained in accordance with the National Institutes of Health Guidelines for the Use of Laboratory Animals. ICR mice were housed in ideal conditions at 20-23°C under a 12-h light/dark cycle with free access to water and food.

### Oocyte Collection and Culture

Germinal vesicle (GV)-intact oocytes were obtained as described previously (37). In brief, female ICR mice were intraperitoneally injected with 5 IU of pregnant mare’s serum gonadotropin (PMSG). Forty-six hours later, the animals were euthanized by cervical dislocation and the ovaries were harvested and transferred to a cell-culture dish containing pre-warmed M2 medium (Sigma, M7167). Cumulus-oocyte complexes were obtained from antral ovarian follicles by manual rupture. Then, denuded GV oocytes were obtained by removing cumulus cells with a narrow-bore glass pipette and gentle washing. For IVM, oocytes were cultured in M16 medium (Sigma, M7292) under paraffin oil for 12 h at 37°C in a 5% CO_2_ incubator. For melatonin treatment, oocytes were cultured in M16 medium at a final concentration of 1 μM melatonin (Sigma, M5250) in accordance with previous studies (38, 39). For IVO, mice injected with PMSG were further injected with 5 IU of human chorionic gonadotropin (hCG) 46 h later. Ovulated MII oocytes were collected from the oviduct ampullae of mice 14 h after hCG injection and cumulus cells were dispersed by brief exposure to M2 medium containing 1 mg/ml of hyaluronidase (Sigma, H3506).

### Library Preparation and Illumina NovaSeq 6000 Sequencing

Total RNA was extracted from 20 MII oocytes after IVM and IVO from three mice of advanced reproductive age reproductively (these were regarded as one group). Three biological replicates obtained *via* both IVM and IVO were used to construct a eukaryotic micro-library. Specifically, we used a SMART-Seq v4 Ultra Low Input RNA Kit (Takara Bio) to obtain full-length cDNA in accordance with the manufacturer’s instructions. cDNA synthesis and amplification were validated by an Agilent 2100 Bioanalyzer (Agilent Technologies) and a High Sensitivity DNA Kit (Agilent Technologies) in accordance with the manufacturer’s instructions. Then, the Nextera XT DNA Library Preparation Kit (Illumina) was used to construct a library that was suitable for sequencing. Finally, a paired-end RNA-seq library was performed on the Illumina NovaSeq 6000 system (2 × 150bp read length).

### DEG Identification and Functional Enrichment Analysis

SeqPrep (https://github.com/jstjohn/SeqPrep) and Sickle (https://github.com/najoshi/sickle) software packages were used to filter the raw paired-end reads with default parameters. The clean reads were then aligned to the mouse reference genome (GRCm39) using the Hisat2 mapping tool (40). The fragments per kilobase per million reads (FPKM) method was then used to calculate gene expression levels; gene abundance was determined by utilizing the RSEM software workflow (41). The analysis of DEGs between the IVM and IVO groups was performed with the R statistical package DESeq2 (42) using |log2FC| ≥ 1 and adjusted *P*-value < 0.05 as thresholds for significance. KEGG pathway enrichment analyses of the identified DEGs were carried out using KOBAS software (43) which incorporates information from the KEGG database (https://www.genome.jp/kegg/).

### The Identification of Alternative Splice Events

All alternative splice (AS) events in the IVM and IVO groups were detected by the replicate multivariate analysis of transcript splicing (rMATS) statistical method (44). Only isoforms that were similar to the reference or consisted of novel splice junctions were considered, and splicing differences were detected in five major forms (exon inclusion, exclusion, alternative 5′, 3′, and intron retention events).

### RNA Extraction, qRT-PCR, and RT-PCR Analysis

Total RNA was extracted from 100 matured oocytes from twelve mice of advanced reproductive age using an RNeasy Micro Kit (Qiagen, 74034) in each group. cDNA was synthesized using the PrimeScript RT Master Mix Kit (Takara, RR036A) and qRT-PCR was performed using the PowerUp SYBR Green Master Mix (Thermo Fisher Scientific, A25742) and the QuantStudio 3 Real-Time PCR System (Applied Biosystems, USA). Relative mRNA levels were normalized to *Gapdh* as a control standard and calculated using the 2^-△△CT^ method. Semi-quantitative RT-PCR was performed using Premix Taq (Takara, RR901A) and specific primers were designed to flank the constitutively expressed exons of differentially spliced genes. PSI values were calculated using the ratio of the band intensity of the exon inclusion divided by the sum of the exon-included and exon-excluded bands, as described previously (45, 46). Primer sequences are given in **Table S1**.

### Detection of Intracellular ROS Levels

For intracellular ROS detection, oocytes were incubated in M2 medium supplemented with 5 μM CM-H2DCFDA (Invitrogen, C6827) at 37°C for 30 min in a 5% CO_2_ atmosphere and a dark environment. Then, the oocytes were washed three times with pre-warmed M2 medium. Then, oocytes from the IVM and IVO groups were placed on glass-bottomed culture dishes and examined with a confocal laser scanning microscope (Carl Zeiss 710, Germany).

### Determination of Adenosine 5′-Triphosphate Content

Adenosine 5′-triphosphate (ATP) content in a pool of 20 oocytes was measured with a bioluminescent somatic cell assay kit (Sigma, USA) as described previously (47, 48). A five-point standard curve (0, 0.1, 0.5, 1.0, 10, and 50 pmol of ATP) was generated in each assay, and ATP levels were calculated by using a formula derived from the linear regression of the standard curve.

### Statistical Analysis

Data are expressed as mean ± standard error of the mean (SEM) from three independent experiments and statistical analyses were performed with GraphPad Prism 5 software (San Diego, CA, USA) and the Student’s *t*-test between the IVM and IVO groups. *P* < 0.05 was considered as statistically significant (*P < 0.05, **P < 0.01, and ***P < 0.001).

## Results

### Sequencing Data Summary

**Figure 1:**
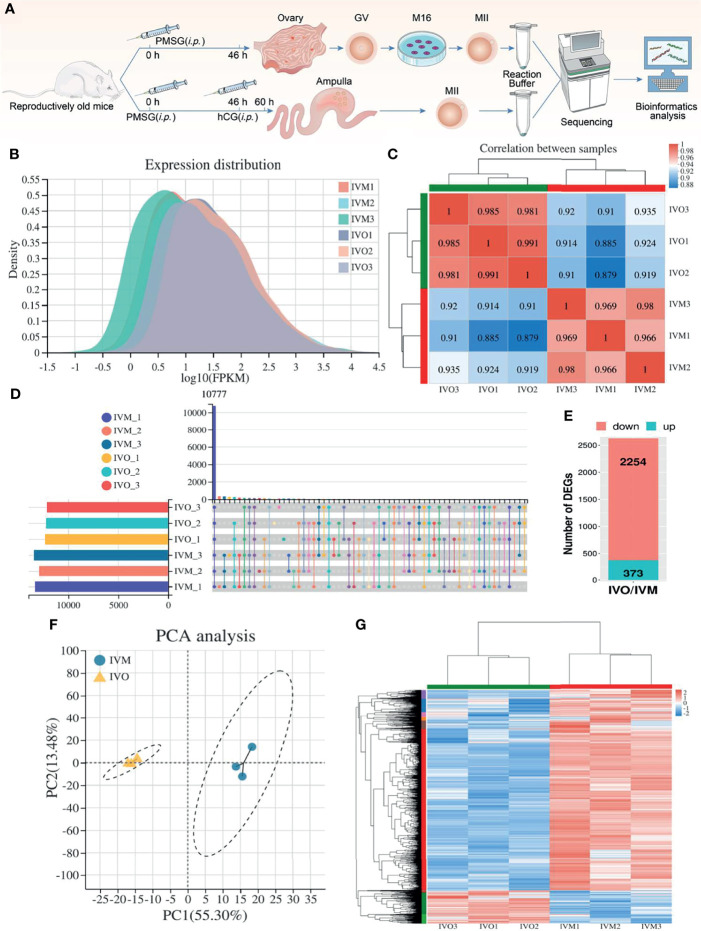
DEG analysis between IVO and IVM groups. **(A)** A schematic showing experimental design and time points. **(B)** Gene expression density distribution in the IVO and IVM groups. **(C)** Correlation analysis between the IVM and IVO groups. The degree of correlation is represented by different colors. **(D)** An UpSet plot summarizing the genes detected in the six groups. **(E)** A stacked column showing the numbers of DEGs in the IVO group compared with the IVM group. **(F)** PCA of gene expression profiles in oocytes between the IVM group (blue dots) and the IVO group (yellow triangles). **(G)** Heatmap showing DEGs; the red rows represent high expression levels and blue rows represent low expression levels. Each column represents an independent sample.

As shown in **Figure 1A**, we used ICR mice at the age of 42-45 weeks (a reproductive advanced age) that were near to the end of their reproductive lifespan and exhibited a decline in oocyte quality, as described in previous studies (49). These mice are commonly regarded as a natural aging model and were used as donors for IVM and IVO oocytes so that we could carry out RNA sequencing. First, we used cDNA to generate libraries for IVM and IVO oocytes, which were then sequenced by the Illumina NovaSeq 6000 platform (three biological replicates per group). Gene expression distribution statistics showed that the six groups of IVM and IVO oocytes from mice of advanced reproductive age presented with similar gene expression distributions (**Figure 1B**). A heatmap for the Pearson’s correlation coefficient indicated that the experiment was reliable, as determined by the fragments per kilobase million (FPKM) value calculated for each sample (**Figure 1C**). A total of 10,777 expressed genes were identified across all six samples, as shown on the UpSet plot (**Figure 1D**). When comparing the IVM and IVO groups, we identified 2,627 DEGs based on specific criteria (|log2FC| ≥ 1 and an adjusted *P*-value < 0.05). Of these, 2,254 genes were down-regulated, and 373 genes were up-regulated in IVO oocytes from mice of advanced reproductive age when compared with IVM oocytes (**Figure 1E**). A list of the DEGs is given in **Supplementary Excel S1**. Furthermore, principal components analysis (PCA) revealed that the DEG clusters of oocytes obtained from mice of advanced reproductive age in the IVO and IVM groups were separated (**Figure 1F**), thus implying that the different culture procedures had a substantial effect on transcriptomic profiles. In addition, we generated a heatmap of DEGs for the IVO and IVM groups using cluster analysis and the FPKM values (**Figure 1G**).

### Functional Analysis of the DEGs

**Figure 2:**
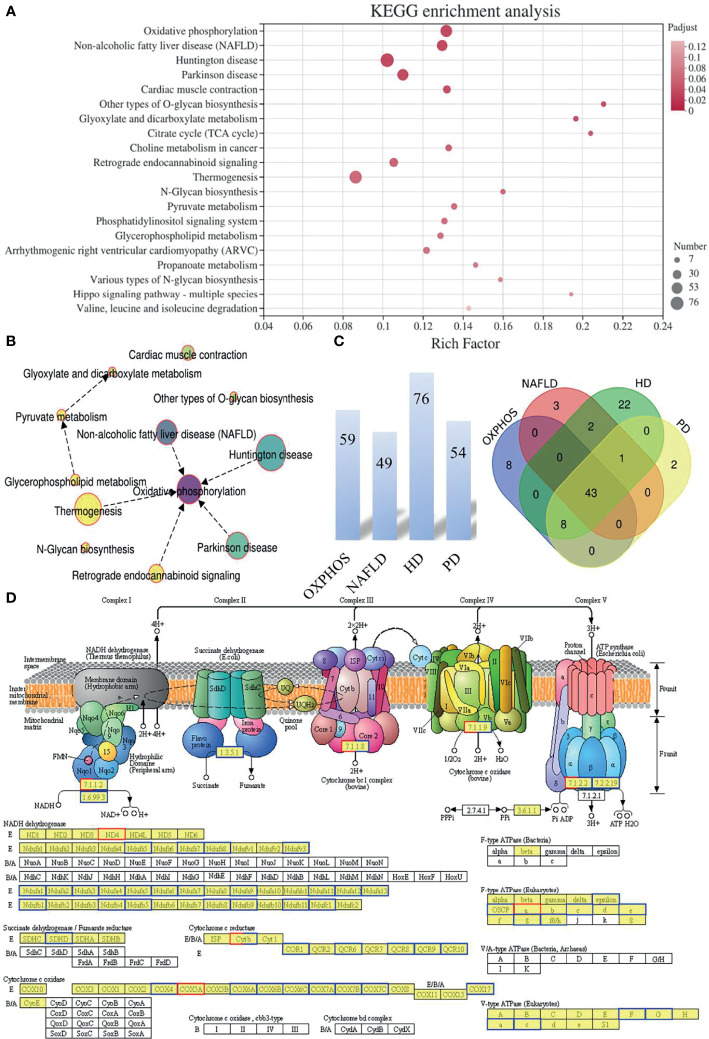
The functional pattern of gene expression between IVO and IVM groups. **(A)** The top 20 enriched KEGG pathways for the DEGs identified between the IVO and IVM groups. The X-axis represents the Rich Factor while the Y-axis represents the names of the pathways. The higher the Rich factor, the higher the degree of enrichment. **(B)** Network chart showing the significantly enriched KEGG pathways. **(C)** The number above each bar indicates the enriched DEGs of the top four terms (the OXPHOS, NAFLD, HD, and PD pathways) and the Venn diagram shows the overlapping DEGs between the top four enriched KEGG pathways. **(D)** KEGG pathway diagram for oxidative phosphorylation. Boxes with red borders represent up-regulated genes while boxes with blue borders represent down-regulated genes when the IVO group was compared with the IVM group.

We mapped the DEGs from mice oocytes of an advanced reproductive age in the IVO and IVM groups to the KEGG pathways database terms in order to interpret the biological consequences of differential expression. KEGG enrichment analysis revealed significant enrichment in terms of OXPHOS pathways (map00190; 59 DEGs; *P*-adjusted = 2.03 × 10^-6^), non-alcoholic fatty liver disease (NAFLD) (map04932; 49 DEGs; *P*-adjusted = 3.11 × 10^-5^), Huntington’s disease (HD) (map05016; 76 DEGs; *P*-adjusted = 2.34 × 10^-4^), and Parkinson disease (PD) (map05012; 54 DEGs; *P*-adjusted = 6.48 × 10^-4^) (**Figure 2A** and **Supplementary Excel S2**). A KEGG enrichment chord plot, featuring the top four terms (the OXPHOS, NAFLD, HD, and PD pathways) suggested that several target genes did not uniquely exist in one single enriched pathway; rather, they were annotated in multiple terms (**Supplementary Figure S1**). A network diagram was constructed to identify significant interaction in the enriched KEGG pathways, which revealed that the OXPHOS pathway was critical (**Figure 2B** **)**. Venn diagram analysis further showed that some of the DEGs were shared among different group combinations. For example, two up-regulated and 41 down-regulated overlapped DEGs were represented in the four KEGG pathways of mitochondrial OXPHOS and diseases related to mitochondria dysfunction (NAFLD, HD, and PD) (**Figure 2C**). In addition, the most significant enrichment pathway diagram (OXPHOS) featured many down-regulated genes in the IVO groups when compared with the IVM group (**Figure 2D**), thus implying that oocytes matured *in vitro* suffer from changes associated with oxidative stress and metabolic activity.

### qRT-PCR Validation of DEGs That Were Enriched in KEGG Pathways

**Figure 3:**
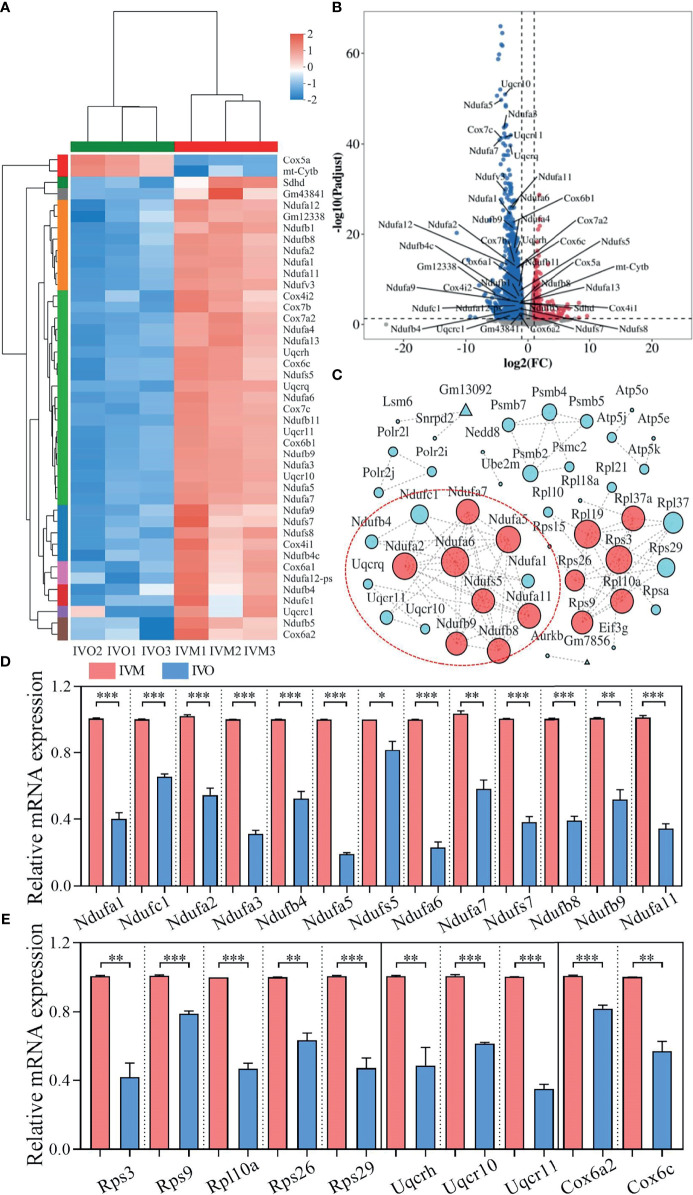
DEG analysis and network patterns for the top four enriched KEGG pathways. **(A)** Heatmap diagram showing the top four enriched KEGG pathways with overlapping DEGs (*P*-adjusted < 0.05 and |log2FC| ≥ 1). **(B)** Volcano plots showing overlapping DEGs in the top four KEGG enrichment analyses. Blue dots indicate down-regulated genes while red dots indicate up-regulated genes. **(C)** A PPI network for the down-regulated genes was constructed using the STRING database to compare the IVO and IVM groups. The node size represents the number of linked lines and connectivity. **(D, E)** The relative mRNA levels of DEGs including NADH-ubiquinone oxidoreductase (complex I) subunits, the ribosomal protein family, ubiquinol-cytochrome c reductase subunits, and cytochrome c oxidase subunits between the IVM and IVO groups, as determined by RNA-seq and confirmed by qRT-PCR assay. Data represent the mean ± SEM of three independent experiments. *P < 0.05, **P < 0.01, and ***P < 0.001.

A heatmap of overlapping DEGs from the top four enriched KEGG pathways showed that 43 protein-encoding genes, including NADH-ubiquinone oxidoreductase (complex I) subunits, cytochrome c oxidase subunits, and ubiquinol-cytochrome c reductase subunits, were significantly altered (**Figure 3A**). Volcano plots were also used to visualize overlapping DEGs, including two up-regulated and 41 down-regulated genes (**Figure 3B**) that met our selection criteria [*P*-adjusted < 0.05 and |log2FC| ≥ 1, as determined by statistical DESeq2 (42)]. To identify relationships between these DEGs, we constructed a protein-protein interaction (PPI) network using the STRING online database (50); this consisted of 48 nodes and 100 edges and featured 2254 down-regulated genes when the IVO groups were compared with the IVM groups (**Figure 3C**). Next, to validate the RNA sequencing data, the relative mRNA levels of DEGs were verified by qRT-PCR and were then classified by function into the NADH-ubiquinone oxidoreductase (complex I) subunits, ribosomal protein family, ubiquinol-cytochrome c reductase subunits, and cytochrome c oxidase subunits (**Figures 3D, E** **)**. Results indicated that data were consistent with those derived from the transcriptomic analysis.

### Supplementation With Antioxidant Melatonin Changed mRNA Levels of the DEGs

**Figure 4:**
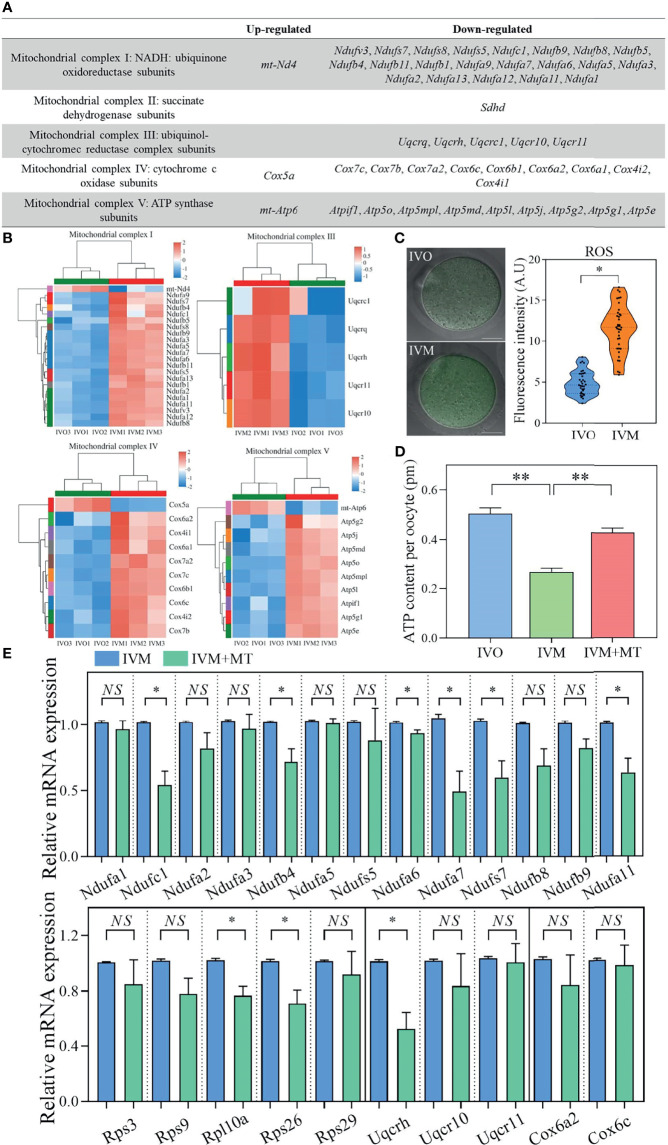
**(A)** List of up- and down-regulated genes in the OXPHOS system of five respiratory chain complexes in the mitochondria when compared between IVO and IVM groups. **(B)** Heatmaps corresponding to the identified DEGs. The red rows represent high expression levels and the blue rows represent low expression levels. Each column represents an independent sample. **(C)** Representative images of ROS signals in IVO and IVM groups. Scale bars = 20 μm. The fluorescence intensity of ROS in IVO and IVM groups. **(D)** Graphs showing the ATP levels in IVO, IVM, and melatonin-treated IVM oocytes (*n* = 20 for each group). **(E)** The relative mRNA levels of target genes including NADH-ubiquinone oxidoreductase (complex I) subunits, the ribosomal protein family, ubiquinol-cytochrome c reductase subunits, and cytochrome c oxidase subunits between the IVM and melatonin-treated IVM oocytes. Data represent the mean ± SEM of three independent experiments. *NS*, not significant, *P < 0.05, and **P < 0.01.

Next, we classified DEGs between the IVO and IVM groups in the OXPHOS system into five respiratory chain complexes within the mitochondria. Analysis showed that respiratory chain complex I had the most DEGs, as shown by a gene list and heatmaps (**Figures 4A, B** **)**. We also measured the ROS levels in IVM and IVO oocytes from mice of advanced reproductive age; the fluorescence intensity of ROS was significantly higher in IVM oocytes compared with IVO oocytes (11.20 ± 1.36; *n* = 38 vs. 4.82 ± 0.60; *n* = 35, *P* < 0.05; **Figure 4C**), thus showing that changes in the expression of OXPHOS pathway genes have an effect on ROS level. The synthesis of ATP through oxidative metabolism was associated with oocyte maturation and the reduced ATP production and increased ROS accumulation are linked to poor oocyte quality according to a previous study (51). These results revealed that the ATP content was lower in IVM oocytes than in IVO oocytes, whereas with melatonin supplementation (1 μM), the ATP generation was restored in the melatonin treated IVM group, as shown in **Figure 4D**. This suggested that IVM-induced mitochondrial dysfunction in reproductively old mice oocytes could be rescued by melatonin treatment. In addition, to ascertain if antioxidant supplementation could change the gene expression profile observed in the IVM group, oocytes were cultured in melatonin supplemented M16 medium; the mRNA expression of genes regulating oxidative phosphorylation were shown to be reduced in oocytes when compared to those without melatonin (**Figure 4E**).

### AS Events Associated With DEGs Detected by RNA-Seq Analysis

**Figure 5:**
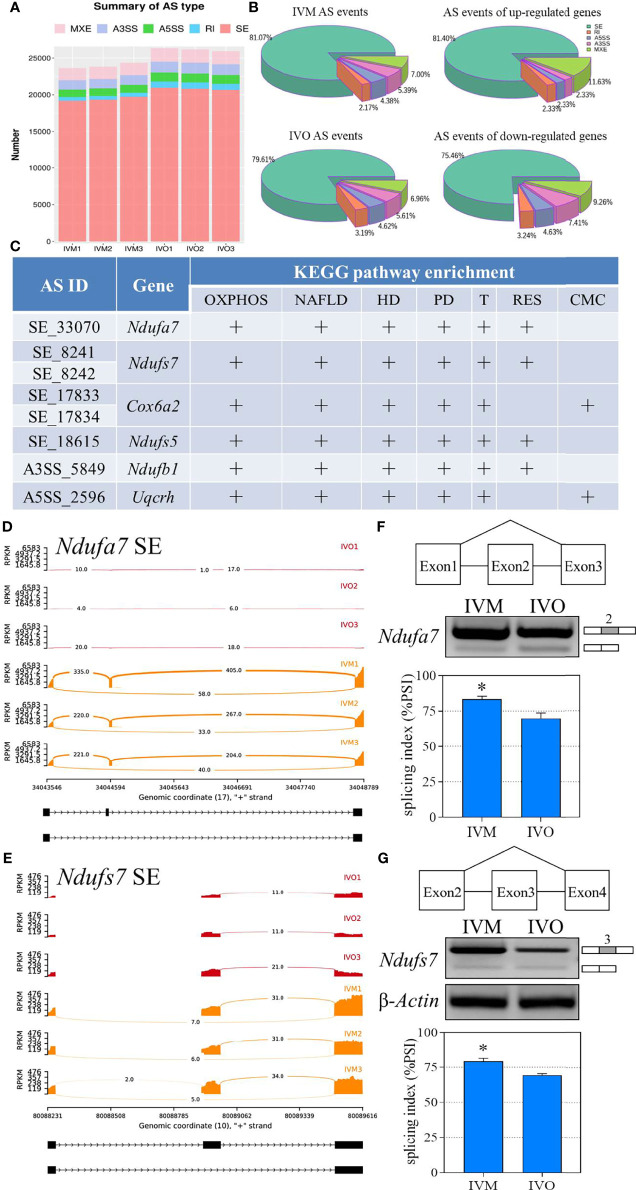
Statistical analysis of the AS events for KEGG enrichment terms. **(A)** A comparison of the number of AS events between the IVO and IVM groups. **(B)** The proportion of AS events of the IVM group, IVO group, the up-regulated and down-regulated genes. **(C)** List of significantly different AS events for the top four KEGG enriched overlapping DEGs (OXPHOS, oxidative phosphorylation; NAFLD, non-alcoholic fatty liver disease; HD, Huntington disease; PD, Parkinson disease; T, Thermogenesis; RES, retrograde endocannabinoid signaling; CMC, cardiac muscle contraction. Sashimi plots depicting the visualize specific splice sites of splicing events for exon skipping of the *Ndufa7* **(D)** and *Ndufs7* **(E)** genes. The red plots represent the IVO group while the orange plots represent the IVM group. The distribution of the number of junction reads is shown for each sample. The schematic in the upper panels represent the different splicing pattern. Representative agarose gels of semi-quantitative RT-PCR for *Ndufa7* exon 2 **(F)** and *Ndufs7* exon 3 **(G)** are shown in the middle panels. β-*Actin* served as an internal control. The histogram in the lower panel shows the average PSI that the IVO oocytes present exon inclusion suppressed compared with the IVM oocytes. Data represent the mean ± SEM of three independent experiments. *P < 0.05.

The mRNA precursors produced by most eukaryotic gene transcription only correspond to a translated protein. However, AS is a process that improves biodiversity by producing mRNA precursors for genes that produce different mRNA splicing isoforms by five different splicing mechanisms under certain conditions: SE, A5SS, A3SS, MXE, and RI (52, 53). We determined the distribution of AS events in the IVM and IVO groups and generated a stacked bar chart that identified SE as the most frequent type of AS event during both *in vivo* and *in vitro* oocyte maturation (**Figure 5A**). In addition, we generated pie charts that illustrated the proportion of each AS event according to the total number of events; SE events represented 81.07% and 79.61% of all AS events in the IVM and IVO groups, respectively (**Figure 5B**). Furthermore, we determined the differential AS events that occurred in genes that were up-regulated in the IVO group. Specifically, there were 35 (81.4%) SE events, five (11.6%) MXE events, one (2.3%) A3SS event, one (2.3%) A5SS event, and one (2.3%) RI event. Then, we determined the differential AS events for the down-regulated genes in the IVO groups. Specifically, there were 163 (75.5%) SE events, 20 (9.3%) MXE events, 16 (7.4%) A3SS events, ten (4.6%) A5SS events, and seven (3.2%) RI events (**Figure 5B**). The different types of AS events corresponding to DEGs including *Ndufa7*, *Ndufs7*, *Cox6a2*, *Ndufs5*, *Ndufb1*, and *Uqcrh* were listed in each enriched KEGG term (**Figure 5C**). These AS events included the SE, A3SS, and A5SS types, thus indicating splicing diversity during oocyte maturation. Next, Sashimi plots were used to visualize the differential splicing patterns with RNA-seq reads coverage across each part of the splice junction between two exons for *Ndufa7* and *Ndufs7* (**Figures 5D, E** **)**. Experimental semi-quantitative RT-PCR validation was performed with specific primers and exon 2 of *Ndufa7* and exon 3 of *Ndufs7* was skipped in the IVO oocytes from mice of advanced reproductive age (**Figures 5F, G** **)**.

## Discussion

The mean age of women at the birth of their first child has increased gradually over the past few decades (54, 55), which has led to a decline in both oocyte quantity and quality. The application of IVM as an ART procedure provides patients with an alternative treatment option that does not involve gonadotrophin stimulation, thus preventing the risk of OHSS and reducing medication costs (56). However, several clinical outcomes, including implantation rates, pregnancy rates, and live birth rates, are known to be lower for IVM than *in vitro* fertilization (57). In the present study, we applied transcriptomic analysis of oocytes derived from mice of advanced reproductive age and identified several DEGs in MII oocytes when comparing between an IVM group and an IVO group. Next, we performed KEGG enrichment analyses to investigate the potential functions of the DEGs. We found that mitochondrial OXPHOS was the most significantly enriched pathway including 59 target genes, followed by NAFLD, HD, and PD. In addition, the OXPHOS is the pathway with the most interactions with other pathways, as visualized by a PPI network.

The OXPHOS pathway is the major source of ATP production in mitochondria and involves five multi-subunit protein complexes that are associated with oocyte maturation (14), fertilization (58), and embryonic development (59). Furthermore, studies have shown that mitochondria are the major source of ROS and, with increasing age, the amount of ROS increases, thus causing cellular damage and a decline in oocyte quality (51). The OXPHOS system contains five respiratory chain complexes within the mitochondria and by analyzing the gene list and a heatmap, we found that respiratory chain complex I had the most DEGs, results of which were similar to a previous study that showed that complexes I and III of the mitochondrial respiratory chain are key contributors to the production of ROS (60, 61). Moreover, several extracellular factors are known to play vital roles in ROS defense during oocyte development *in vivo*. For example, follicular fluid has the ability to prevent the harmful effects of ROS due to the involvement of redox reactions (62, 63), and the enclosed cumulus cells act as scavengers by protecting oocytes against ROS toxicity (63, 64). In addition, the influence of physical factors, such as exposure to cool white or warm white fluorescent lights, can promote the abnormal accumulation of ROS in the development of mammalian zygotes (65). This explains why the levels of ROS are increased in IVM-derived oocytes due to lack of *in vivo* milieu (66); this concurs with the findings of the present study. Furthermore, several previous publications have shown that antioxidant supplementation of the IVM medium significantly reduced the levels of intracellular ROS, thus enhancing oocyte maturation rate and increasing the quality of oocytes from mice of advanced reproductive age (67, 68). In our study, we demonstrate that antioxidant melatonin supplementation may reduce the gene expression profile of the OXPHOS pathway and resolve the problem of insufficient ATP production in the IVM group.

We next constructed a PPI network for down-regulated genes and identified several genes that exhibited strong associations with ribosomal protein family genes, including *Rpl10a*, *Rpl19*, *Rps3*, *Rpl37a*, *Rps9*, and *Rps26*, that presented strong associations with hub nodes. A previous study showed that Rps26 acts as a key regulator during follicular development and oocyte growth by controlling histone methylation and mRNA synthesis activity; these are key mechanisms for female fertility (69). Recently, Peng et al. reported that *RpS3* deficiency leads to a lower blastocyst formation rate and causes early embryonic arrest (70). Collectively, the identification of these related genes and enriched pathways has highlighted the potential mechanisms underlying the poor clinical outcomes and embryonic development associated with the IVM procedure and identify new therapeutic molecular targets.

AS is a complex process that enables genome complexity and biological diversity among eukaryotes; these events occur in approximately 95% of multi-exon genes in humans (25). We investigated the functionality of AS events in IVM and IVO MII oocytes from mice of advanced reproductive age using RNA-seq analysis and found that SE was the predominant form of AS event. Previous studies have revealed that AS events are associated with meiosis in mouse oocytes and that the depletion of SRSF3 results in meiotic resumption defects due to the dysregulation of *Brd8* and *Pdlim7* exon inclusion (33). The knockdown of *Esrp1* induces female infertility and oocyte meiotic arrest by affecting pre-mRNA splicing alterations involving *Lsm14b*, *Trb53bp1*, and *Rac1* (34). In our study, the DEGs between the IVO and IVM groups included *Ndufa7*, *Ndufs7*, *Cox6a2*, *Ndufs5*, *Ndufb1*, and *Uqcrh*, all of which were enriched in the mitochondrial OXPHOS pathway involving SE, A3SS, or A5SS splicing events, and overlapped with non-alcoholic fatty liver disease, Huntington disease, Parkinson disease, and thermogenesis-enriched pathways. Recent research has shown that the knockout of *UQCRH* reinforces the Warburg effect (WE) (71) and the WE plays a critical role in embryogenesis by supporting rapid cell proliferation (72). Previously, our group reported that calcium signaling was inhibited in IVM oocytes but not IVO oocytes in humans (22) and calcium treatment leads to a decreased content of NDUFS7 in the mitochondria (73); this might explain the fact that our transcriptomic analysis found a reduction of *Ndufs7* in the IVO group. Future research needs to investigate AS events in these DEGs by either knockdown or exogenous overexpression experiments. In the present study, we also found that the *Zfp982* gene undergoes the most frequent AS events, including SE, MXE, A3SS, and RI events (**Supplementary Figure S2**). Dehghanian et al. reported that *Zfp982* acts as a transcription factor by interacting with YAP1 to maintain stemness in mouse embryonic stem cells (74). Furthermore, *Obox1* as an oocyte-specific factor can promote induced pluripotent stem cells induction by regulating mesenchymal-to-epithelial transition during reprogramming by inhibiting cell-cycle-related gene expression (75). Little is known about AS events in oocyte maturation; consequently, the alternative RNA splicing genes identified in this study deserve further to be investigated, particularly with regards to their mechanisms of action.

In the present study, we systematically compared the expression profiles of DEGs between groups of IVM and IVO oocytes from mice of advanced reproductive age. We identified the potential functions of these DEGs and used KEGG enrichment and PPI network analysis to determine that OXPHOS was the most significantly enriched and interactive pathway. Heatmaps of the five respiratory chain complexes, particularly complex I, indicated the potential involvement of specific antioxidants. Notably, the AS events detected in the present study demonstrated the diversity of post-transcriptional gene regulation in MII oocytes when cultured in different processes. Therefore, the identified key genes may represent potential therapeutic targets for the treatment of infertility in women of advanced reproductive age.

## Data Availability Statement

The datasets presented in this study can be found in online repositories. The names of the repository/repositories and accession number(s) can be found below: https://www.ncbi.nlm.nih.gov/geo/, GSE182711.

## Ethics Statement

The animal study was reviewed and approved by the Institutional Animal Care and Use Committee of Peking University.

## Author Contributions

JQ designed and supervised the study. HQ performed the experimental work and data analysis with help from YQ. HQ drafted the manuscript with support from YQ, RL, and JQ. JQ, HQ, and YQ provided the funding. All authors contributed to the article and approved the submitted version.

## Funding

This project is funded by the China Postdoctoral Science Foundation (nos. 2019TQ0009 and 2020M670060), the National Key Research and Development Program of China (no. 2019YFA0801400), the National Natural Science Foundation of China (nos. 81521002, 81730038, and 31801244), and CAMS Innovation Fund for Medical Sciences (no. 2019-I2M-5-001).

## Conflict of Interest

The authors declare that the research was conducted in the absence of any commercial or financial relationships that could be construed as a potential conflict of interest.

## Publisher’s Note

All claims expressed in this article are solely those of the authors and do not necessarily represent those of their affiliated organizations, or those of the publisher, the editors and the reviewers. Any product that may be evaluated in this article, or claim that may be made by its manufacturer, is not guaranteed or endorsed by the publisher.

## Supplementary Material

Supplementary Figure 1 The chord diagram represents DEGs associated with the top four enriched KEGG pathways (the OXPHOS, NAFLD, HD, and PD) that are shown on the right. The color links merge genes with the log fold change are located on the left.

Supplementary Figure 2
**(A)** List of genes undergoing three or more AS events, including *Zfp982*, *Pbdc1*, *Obox1*, *Phf20*, *Tnfsf13b*, *Platr30*, *Scml2*, and *Trmt11*. **(B)** List of genes undergoing six or more AS events. **(C)** The top 20 enriched KEGG pathways for differential AS events of the DEGs between the IVO and IVM groups.
