# Supplementary figures and images for: In Vivo and In Vitro Matured Oocytes From Mice of Advanced Reproductive Age Exhibit Alternative Splicing Processes for Mitochondrial Oxidative Phosphorylation
Source: Front Endocrinol (Lausanne). 2022 Jan 26;13:816606. doi: 10.3389/fendo.2022.816606 (PMC8826577; doi:10.3389/fendo.2022.816606)

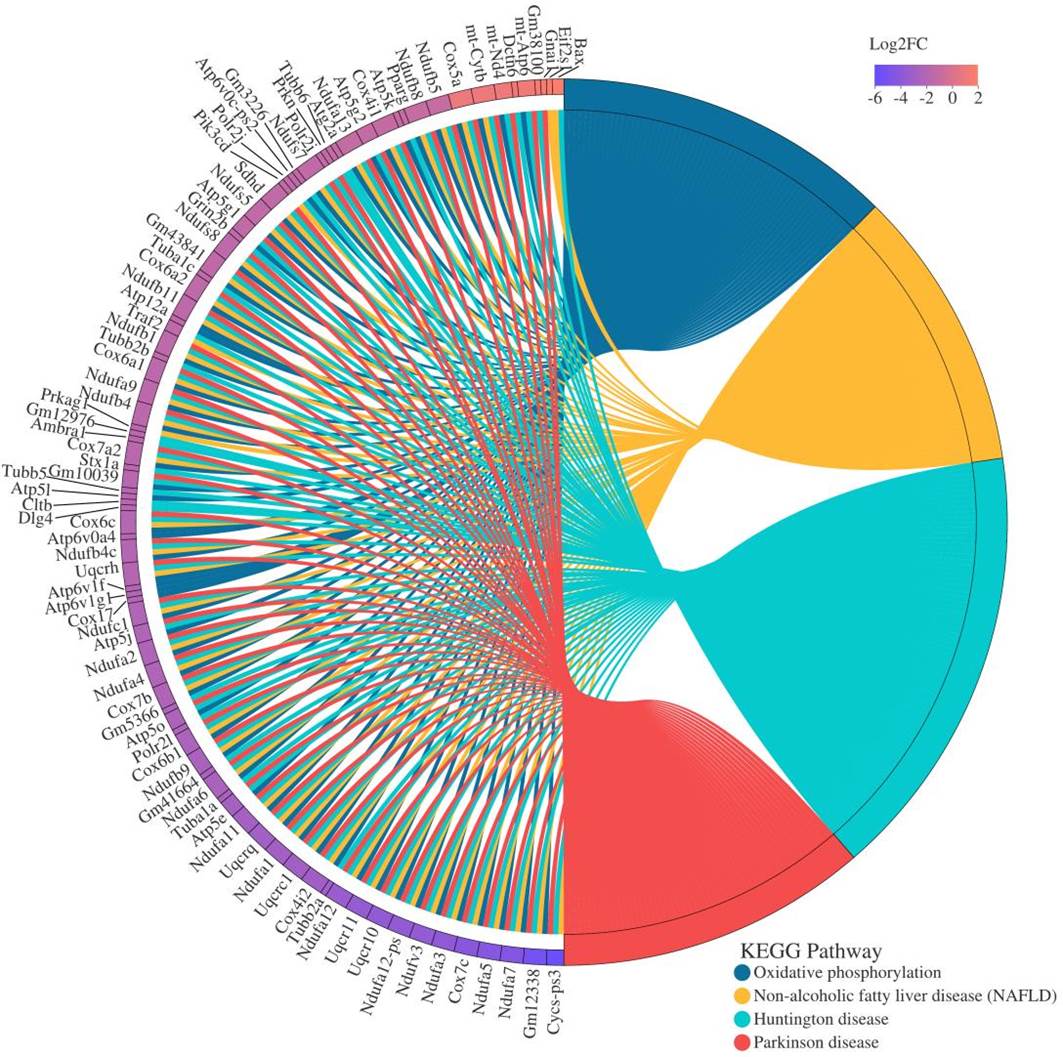

Supplement: Supplementary Figure 1 — The chord diagram represents DEGs associated with the top four enriched KEGG pathways (the OXPHOS, NAFLD, HD, and PD) that are shown on the right. The color links merge genes with the log fold change are located on the left. [file Image_1.jpeg]

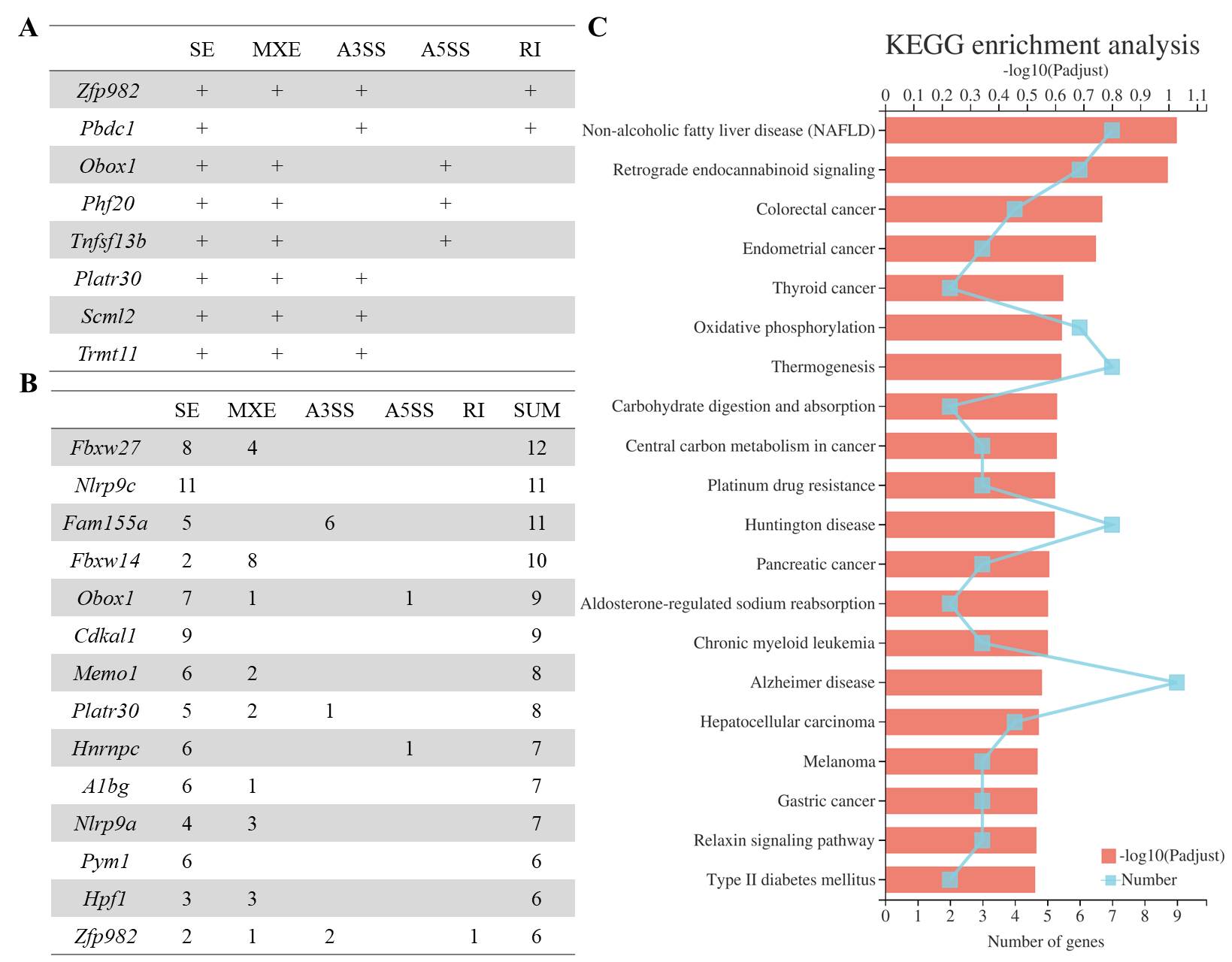

Supplement: Supplementary Figure 2 — (A) List of genes undergoing three or more AS events, including Zfp982, Pbdc1, Obox1, Phf20, Tnfsf13b, Platr30, Scml2, and Trmt11. (B) List of genes undergoing six or more AS events. (C) The top 20 enriched KEGG pathways for differential AS events of the DEGs between the IVO and IVM groups. [file Image_2.jpeg]
