# Supplementary material for: In Vivo and In Vitro Matured Oocytes From Mice of Advanced Reproductive Age Exhibit Alternative Splicing Processes for Mitochondrial Oxidative Phosphorylation
Source: Front Endocrinol (Lausanne). 2022 Jan 26;13:816606. doi: 10.3389/fendo.2022.816606 (PMC8826577; doi:10.3389/fendo.2022.816606)
Supplement: Supplementary file 5 [file Table_3.docx]

| **Quantitative RT-PCR** | | |
| --- | --- | --- |
| **Gene name** | **Forward primer sequence** | **Reverse primer sequence** |
| *Ndufa1* | 5’-ATGTGGTTCGAGATTCTCCCT-3’ | 5’-TTTGTGGATGTACGCAGTGGA-3’ |
| *Ndufc1* | 5’-GTAGTGCTGCGCTCGTTTTC-3’ | 5’-CCAACCAGTTAGGTTTGGCAT-3’ |
| *Ndufa2* | 5’-TTGCGTGAGATTCGCGTTCA-3’ | 5’-ATTCGCGGATCAGAATGGGC-3’ |
| *Ndufa3* | 5’-ATGGCCGGGAGAATCTCTG-3’ | 5’-AGGGGCTAATCATGGGCATAAT-3’ |
| *Ndufb4* | 5’-CTTGATTCGCTGGACCTATGC-3’ | 5’-GGAGTGGGCCTGAAATTAGGA-3’ |
| *Ndufa5* | 5’-AGCTGGATATGGTCAAGGCG-3’ | 5’-GCCACTTCCACTGGTTAGCA-3’ |
| *Ndufs5* | 5’-ATGCCTTTCCTTGACATACAGAAAA-3’ | 5’-CACCGAGCGGCGTTCTTAT-3’ |
| *Ndufa6* | 5’-CACACGTTATGCGGTTTTTCC-3’ | 5’-GGGTCATGGCCCATATAGAACT-3’ |
| *Ndufa7* | 5’-TCCGCTACTCGCGTTATCCA-3’ | 5’-GATTGAGGGAGGCACAACTTC-3’ |
| *Ndufs7* | 5’-CGCTCCGAAAGGTGTACGAC-3’ | 5’-ACCGAGTAGGAGTAGTGGTAGT-3’ |
| *Ndufb8* | 5’-TGTTGCCGGGGTCATATCCTA-3’ | 5’-GCATCGGGTAGTCGCCATAC-3’ |
| *Ndufb9* | 5’-GGTACTTTGCTTGCTTGATGAGA-3’ | 5’-TGGGAAGATATACGGCTGAGG-3’ |
| *Ndufa11* | 5’-CGGCATTTGCGGCATAATCG-3’ | 5’-GACACAGGTGGTAAGGCCAA-3’ |
| *Cox6a2* | 5’-CTGCTCCCTTAACTGCTGGAT-3’ | 5’-GATTGTGGAAAAGCGTGTGGT-3’ |
| *Cox6c* | 5’-ATGAGTTCCGGTGCGCTGTTGCCCA-3’ | 5’-GAACCCGCAGACGCTTGG-3’ |
| *Rps3* | 5’-AAGTCCGAGTTACACCAACCA-3’ | 5’-TCTCTGATCCGACGACCCTTC-3’ |
| *Rps9* | 5’-TTGTCGCAAAACCTATGTGACC-3’ | 5’-GCCGCCTTACGGATCTTGG-3’ |
| *Rpl10a* | 5’-ATGAGCAGCAAAGTCTCACG-3’ | 5’-TGAGGGTCGTAGTTCTTCAGG-3’ |
| *Rps26* | 5’-TCATTCGGAACATTGTAGAAGCC-3’ | 5’-ACAGCTCACGCAATAATGCAG-3’ |
| *Rps29* | 5’-GTCTGATCCGCAAATACGGG-3’ | 5’-AGCCTATGTCCTTCGCGTACT-3’ |
| *Uqcrh* | 5’-GTGGACCCCCTAACAACAGTG-3’ | 5’-CGGGAAGACACGCGATTATCA-3’ |
| *Uqcr10* | 5’-ATCCCTTCGCGCCTGTACT-3’ | 5’-GTGCTCGTAGATCGCGTCT-3’ |
| *Uqcr11* | 5’-ATGCTGAGCAGGTTTCTAGGCCCGC-3’ | 5’-CCGGCTGTGGGAATCCAGTTT-3’ |
| *Gapdh* | 5’-AGGTCGGTGTGAACGGATTTG-3’ | 5’-TGTAGACCATGTAGTTGAGGTCA-3’ |
| **Semi-quantitative RT-PCR** | | |
| **Gene name** | **Forward primer sequence** | **Reverse primer sequence** |
| *Ndufa7* | 5’-GGCGTCCGCTACTCGCGTTA-3’ | 5’-GACATGATGATTGAGGGAGG-3’ |
| *Ndufs7* | 5’-CGGCTCCTGGCCTGCTCTCT-3’ | 5’-CCCAGTTGATGAGGTCATCC-3’ |
| β*-Actin* | 5’-AGATCAAGATCATTGCTCCTCCTGA-3’ | 5’-GCAGCTCAGTAACAGTCCGC-3’ |
